# ANGER, LOVE, SADNESS: DO EMOTION WORDS HAVE THE SAME SEMANTIC MEANING ACROSS AGE-GROUPS?

**DOI:** 10.1093/geroni/igz038.3099

**Published:** 2019-11-08

**Authors:** Madeline J Nichols, Jennifer A Bellingtier, Frances Buttelmann

**Affiliations:** 1 Furman University, Greenville, South Carolina, United States; 2 Friedrich Schiller University Jena, Jena, Germany; 3 Friedrich-Schiller-Universität, Jena, Thüringen, Germany

## Abstract

Every day we use emotion words to describe our experiences, but past research finds that the meanings of these words can vary. Furthermore, historical shifts in language use and experiential knowledge of the emotions may contribute to age-differences in what these emotion words convey. We examined age-related differences in the valence, arousal, and expression connoted by the words anger, love, and sadness. We predicted age-related differences in the semantic meanings of the words would emerge such that older adults would more clearly differentiate the positivity/negativity of the words, whereas younger adults would report higher endorsement for the conveyed arousal and expression. Participants included American and German older adults (N=61; mean age=68.98) and younger adults (N=77; mean age=20.77). Using the GRID instrument (Swiss Center for Affective Sciences, 2013), they rated each emotion word for its valence, arousal, and expression when used by a speaker of the participant’s native language. Across emotions and dimensions, older adults were generally more moderate in their understanding of emotion words. For example, German older adults rated anger and sadness as suggesting the speaker felt less bad and more good than the younger adults. American older adults rated love as connoting the speaker felt more bad and less good than younger adults. Arousal ratings were higher for German younger, as opposed to older, adults. Cultural differences were most pronounced for sadness such that German participants gave more moderate answers than American participants. Overall, our research suggests that there are age-related differences in the understanding of emotion words.

